# Green tea extract attenuates CCl4-induced hepatic injury in male hamsters via inhibition of lipid peroxidation and p53-mediated apoptosis

**DOI:** 10.1016/j.toxrep.2015.08.001

**Published:** 2015-08-10

**Authors:** Rania Abdel Rahman Elgawish, Haidy G. Abdel Rahman, Heba M.A. Abdelrazek

**Affiliations:** a Department of Forensic Medicine and Toxicology, Faculty of Veterinary Medicine, Suez Canal University, Ismailia, Egypt; b Department of Clinical Pathology, Faculty of Veterinary Medicine, Suez Canal University, Ismailia, Egypt; c Department of Physiology, Faculty of Veterinary Medicine, Suez Canal University, Ismailia, Egypt

**Keywords:** Green tea, CCl4, Hepatotoxicity, p53, Lipid profile

## Abstract

Keeping in mind the beneficial effects of GTE administration on liver damage, the present study was undertaken to evaluate the hepatoprotective effect of green tea extract (GTE) against carbon tetrachloride (CCl4)-induced liver injuries in male hamsters for 8 weeks. Twenty hamsters were equally divided into 4 groups, the control ones (group I) received only dis. water. Hamsters of group II had free access to 10% of GTE, while hamsters of group III received 1 ml/kg of 50% CCl4 in corn oil via gavage daily. Hamsters of group IV (GTE + CCl4) received a free access to GTE supplementation in combination with 1 ml/kg of 50% CCl4 in corn oil via gavage daily. Lipid profile, hepatic enzyme levels and apoptosis molecular marker (p53) were investigated in hamsters. GTE + CCl4 treated hamsters showed lower levels of hepatic malondialdehyde (MDA) than CCl4 exposed hamsters. Hepatic activity levels of GSH, ALD and cytochrome 450 reductase were declined after CCl4 administration while they were remarkably improved with GTE administration. Serum lipid profiles as T-cholesterol (TC), triglyceride (TG) and low density lipoproteins (LDL) were improved in GTE and CCl4 treated hamsters than CCl4 group. Moreover, hepatic tissue damage and p53 expression induced with CCl4 were improved with the treatment of GTE. These results suggested that GTE possesses hepatoprotective properties against the effect of CCl4.

## 1 Introduction

CCl4 is a widely used industrial solvent and it is the best-characterized animal model of xenobiotic-induced, oxidative stress-mediated hepatotoxicity [36], [25]. CCl4 induces the production of several types of reactive oxygen species (ROS), thereby causing liver injury [45]. These ROS can bind to polyunsaturated fatty acids, forming alkoxy and peroxyl radicals to produce lipid peroxide, causing cell membrane damage and changes in enzyme activity [52]. Oxygen-derived free radicals and lipid peroxidation play a critical role in the pathogenesis of various liver diseases including hepatic fibrosis [41], [31], hepatic injury, apoptosis, and necrosis [56], [29]. Accordingly, elimination of free radicals and prevention of lipid peroxidation have been targeted in prevention and treatment of hepatic damage.

DNA fragmentation induces p53 gene expression, blocks cells in the G-phase of the cell cycle, while severe DNA damage triggers apoptosis [27]. p53 activation enhances X-Inhibitors of apoptosis protein (X-IAP) inhibition-induced cell death by promoting mitochondrial release of second mitochondria-derived activator of caspases [8] thus resulting in apoptosis [14], [44], [42]. Therefore, inhibiting p53-dependent hepatocyte apoptosis may be an effective therapeutic strategy for the treatment and prevention of hepatic fibrosis.

More attention has been paid to the protective effects of natural antioxidants against chemically induced toxicities [17]. It has been established that curcumin, α-lipoic acid, and *N*-acetylcysteine exert multiple pharmacological actions that involve antioxidant activities and thus suppress fibrogenesis in rats with CCl4-induced liver injury [19], [33], [54]. In addition, rutin, flavonoid glycosides, possess hepatoprotection against CCl4-induced liver injuries [28]. Green tea prevents oxygen free radical-induced hepatocyte lethality and inhibits carcinogen or toxin induced liver oxidative DNA damage [7], [26]. The protective effects of green tea extracts (GTE) against liver fibrosis and liver cirrhosis have been reported in rats [6], mice [10] and hamsters [11]. Although green tea has traditionally been considered safe, emerging reports linking liver injury, and in some cases liver failure, with the use of green tea extract should not be ignored [40]. Moreover, Teschke et al. [46] concluded that the published hepatotoxicity case reports in connection with the use of GTE provide no clinical evidence that GTE may increase the risk of liver injury by drugs, although partial inhibition of human hepatic and intestinal microsomal cytochromes by GTE was observed *in vitro*.

Information of the effect of green tea extract on CCl4-induced hepatic injury in male hamsters is scarce. The present study was designed to investigate the physiochemical effects of GTE on activities of hepatic lipid peroxidation, antioxidant enzymes, alcohol dehydrogenase, cytochrome P450 reductase,lipid profiles and hepatic cell apoptosis as markers for hepatic injury in hepatotoxic hamster model induced by CCl4.

## 2 Materials and methods

### 2.1 Chemicals and green tea extract

Carbon tetrachloride was provided from AlGomhoria Co., (Egypt). GTE was prepared as described by Babu et al. [3]. Briefly, green tea leaves were minced in a miller and tea powder was extracted with 95% methanol (1:10 w/v) for 2 days with constant stirring. Suspensions were filtered to retain the clear solution. The residue was extracted again. The pooled tea extract was vacuum evaporated below 50 °C. The dried extracts were stored at 4 °C [1]. The solution was made by soaking 100 g of the extract in 1 L of boiling dis. water for 5 min. to make 10% of GTE. This solution was provided to hamsters as their sole source of drinking water.

### 2.2 Animals

A total of 20 male hamsters weighing 75–120 g body weight were purchased from Laboratory Animal House of Faculty of Veterinary Medicine, Suez Canal University, Egypt. They were kept for 2 weeks to be acclimatized before the commencement of the experiment. All the hamsters were caged with saw dust covered floor, in a quiet and temperature controlled room (23 ± 4 °C). The hamsters were given free access to standard diet. All the protocols regarding this study were approved by institutional ethical committee and conducted according to the ethical guidelines for the use of animals in laboratory experiments of the Faculty of Veterinary Medicine, Suez Canal University, Egypt.

### 2.3 Experimental design

The hamsters were randomly divided into 4 experimental groups consisting of 5 hamsters per group: Group I, was considered as control hamsters, fed on standard diet and dis. water. Group II, contained hamsters that had free access to 10% GTE prepared in dis. water, the volume of GTE consumed by each hamster was measured every morning, and the mean intake of GTE was calculated. Group III, contained hamsters treated with CCl4 (1 ml/kg b.w., of 50% solution prepared in corn oil), the dose was given via gastric tube every day for 8 weeks. Group IV contained hamsters that received CCl4 (1 ml/kg b.w., of 50% solution prepared in corn oil), daily for 8 weeks as well as free access to GTE (10%).

### 2.4 Body weight gain and green tea intake

Body weight gain was recorded by subtracting the final body weight from the initial body weight. Also free access to GTE (10%) and the mean intake of GTE/ hamster was calculated daily.

### 2.5 Blood sampling

After 8 weeks treatments, anaesthetised fasted overnight animals were decapitated and blood samples were collected in sterilized plain tubes. Serum was stored at −20 °C for determination of lipid profile.

### 2.6 Tissue sampling

Liver was excised, rinsed with ice phosphate buffer saline, dried by filter paper and weighed. Liver for each hamster was divided into 5 parts; 4 of which were kept at −80 °C until preparation of liver homogenate for malondialdehyde, reduced glutathione, alcohol dehydrogenase and cytochrome P450 reductase levels assay. The remaining part was immersed in 10% neutral buffered formalin for histopathological and immunohistochemical examination.

### 2.7 Estimation of malondialdehyde (MDA)

The MDA content in liver homogenate was assayed calorimetrically at wave length of 532 nm using commercial kit (BioVision, USA) according to the method described by Ohkawa et al. [37].

### 2.8 Reduced glutathione activity (GSH)

GSH activity in liver homogenate was determined at absorbance 412 nm using calorimetric kit (BioVision, USA) according to the method described by Tietez [47].

### 2.9 Alcohol dehydrogenase (ADH)

Alcohol dehydrogenase activity in liver homogenate was determined by calorimetric kit (GenWay Biotech. Inc., USA) at absorbance of 450 nm.

### 2.10 Cytochrome P450 reductase

Cytochrome P450 reductase in liver homogenate was estimated using ELISA Kit (Life Science Inc., China) at absorbance of 450 nm.

### 2.11 Lipid profile

Serum levels of high-density lipoprotein (HDL) cholesterol (Stanbio Laboratory, USA), total cholesterol (TC) (ELITech Diagnostic, France) and triglycerides (TG) (ELITech Diagnostic, France) were measured using enzymatic calorimetric kits according to Tietz [48]. Plasma low density lipoprotein cholesterol was calculated by Friedwald formula described by Davidson and Rosenson [13].

### 2.12 Histopathology

Formalin fixed liver sections were prepared using standard procedures for Hematoxylin and Eosin stain as described by Bancroft et al. [4]. Moreover, five-micron sections of livers were stained with Masson’s trichrome for hepatocyte fibrosis and examined under the microscope.

### 2.13 Immunohistochemistry of p53

The paraffin embedded livers were cut into 5 μm sections and mounted on positively charged slides for p53 immunohistochemistry according to Chang et al. [9]. Sections were deparaffinized in xylene and rehydrated with a descending series of absolute ethanol, followed by water. The sections were incubated with Primary monoclonal antibody for p53 D07 (NCL-L-p53-DO7; Novocastra, Newcastle, UK) at a dilution of 1:200, for 2 h at 25 °C in a humidification chamber. The slides were washed three times for 3 min. each with PBS. Biotinylated polyvalent secondary antibody (Thermo Scientific Co., UK) was applied to tissue sections and co-incubated for 30 min. The reaction was visualized by Metal Enhanced DAB Substrate Working Solution to the tissue and incubated 10 min after washing. Counterstaining was performed by adding adequate amount of hematoxylin stain to the slide to cover the entire tissue surface. For quantitative analysis, the intensity of immunoreactive parts was used as a criterion of cellular activity after subtracting background noise. Measurement was done using an image analyzer (Image J program). From each slide of both experimental groups, 7 fields were randomly selected. The total field and immunohistochemial (IHC) stained areas were calculated and the percentage of IHC stained area calculated as follow: %IHC stained area = IHC stained area/total area X 100.

### 2.14 Statistics

Differences of green tea intake between GTE and CCl4 + GTE groups were evaluated by student *t*-test. One-way analysis of variance (ANOVA) was used for examining differences among groups followed by Tukey’s post hoc test. A *P* value of <0.05 was considered to indicate significance. All the analyses were done using GraphPad Prism (Version 5.01, GraphPad Software, San Diego, USA).

## 3 Results

### 3.1 Body weight gain, liver weights and green tea intake

Body weight gain, relative liver weight and GTE intake of hamsters did not differ significantly between control and treated hamsters (Table 1).

**Table 1:** Changes in body, absolute and relative liver weights (mean ± SEM) of control and hamsters treated with GTE, CCl4 and GTE with CCl4 after 8 weeks of treatment

Parameters	Control	GTE	CCL4	GTE+CCl4
Body weight gain (g)	5.3 ± 6.9	23.3 ± 4.6	9.5 ± 9.8	17.5 ± 4.5
Absolute liver weight (g)	3.7 ± 0.1 ^a^	5.0 ± 0.5 ^ab^	5.4 ± 0.5 ^b^	6.4 ± 0.4 ^b^
Relative liver weight (Abs g/ g b.w. x 100)	4.6 ± 0.2	4.8 ± 0.4	4.8 ± 0.1	5.3 ± 0.2
GTE intake (ml/ day)		26.3 ± 0.6		27.3 ± 0.7

Different superscripts within the same row means significant at *P *< 0.05.

### 3.2 Lipid peroxidation

Lipid peroxidation was measured as MDA in the experimental hamsters. MDA of CCl4-treated hamsters was significantly (*P* < 0.001) increased compared to that in control group, MDA level of GTE supplementation in combination with CCl4 was significantly (*P* < 0.01) lowered than CCl4 group (Table 2).

**Table 2:** GSH, MDA, ALD and cytochrome 450 (mean ± SEM) of control and hamsters treated with GTE, CCl4 and GTE with CCl4 after 8 weeks of treatment.

Parameters	Control	GTE	CCL4	GTE+CCl4	*P*
GSH (mg/g)	20.7 ± 0.1 ^a^	20.6 ± 0.2 ^^a^^	14.9 ± 0.2 ^^b^^	17.5 ± 0.3 ^^c^^	*P* < 0.001
MDA (nmol/g)	0.5 ± 0.02 ^^a^^	0.5 ± 0.01 ^^a^^	0.9 ± 0.02 ^b^	0.8 ± 0.02 ^c^	a vs b *P* < 0.001
					b vs c *P* < 0.01
ALD (U/g)	28.8 ± 0.4 ^^a^^	28.7 ± 0.2 ^a^	22.8 ± 0.7 ^b^	25.7 ± 0.6 ^c^	a vs b *P* < 0.001
					a vs c *P* < 0.05
					b vs c P < 0.05
Cytochrome P450 reductase (ng/g)	3.9 ± 0.07 ^a^	4.1 ± 0.06 ^a^	1.9 ± 0.08 ^b^	3.0 ± 0.07 ^c^	*P* < 0.001

### 3.3 Reduced glutathione, alcohol dehydrogenase and cytochrome P450 reductase

Activity levels of GSH, ALD and cytochrome P450 reductase were declined significantly (*P* < 0.001) after CCl4 treatment. Interestingly, the activities of these enzymes were significantly (*P* < 0.05) improved with GTE in CCl4 treated hamsters (Table 2).

### 3.4 Lipid profiles

In the CCl4 treated hamsters, the serum cholesterol (TC), triglyceride (TG) and low density lipoprotein (LDL) levels were increased by 22%, 21%, and 57%, respectively, while high density lipoprotein (HDL) concentration was decreased by 36% compared to that in control hamsters. Interestingly, GTE supplementation in combination with CCl4 improved the lipid profile in treated hamsters (Table 3).

**Table 3:** Changes of total cholesterol (TC), high density lipoprotein (HDL), triglyceride(TG) and low density lipoprotein (LDL) (mean ± SEM) in control and treated hamsters with GTE, CCl4 and GTE with CCl4 after 8 weeks of treatment.

Parameters	Control	GTE	CCL4	GTE + CCl4	*P*
TC(mg/dl)	127.8 ± 6.2 ^ab^	103.0 ± 1.6 ^a^	157.0 ± 11.2 ^b^	140.0 ± 10.7 ^bc^	a vs b *P* < 0.01
					a vs bc *P* < 0.05
HDL(mg/dl)	36.0 ± 1.1^ac^	62.3 ± 6.1 ^^b^^	23.0 ± 3.1^c^	29.5 ± 2.4 ^c^	ac vs b *P* < 0.01
					b vs c *P* < 0.001
TG(mg/dl)	158.3 ± 11.7	117.8 ± 8.5	251.8 ± 47.4	232.5 ± 52.6	NS
LDL(mg/dl)	45.8 ± 6.8^ac^	29.0 ± 5.1 ^a^	68.8 ± 4.4 ^b^	62.5 ± 3.4 ^^bc^^	ac vs b *P* < 0.05
					a vs b *P* < 0.0001
					a vs bc *P* < 0.01

### 3.5 Liver histopathology

**Fig. 1:**
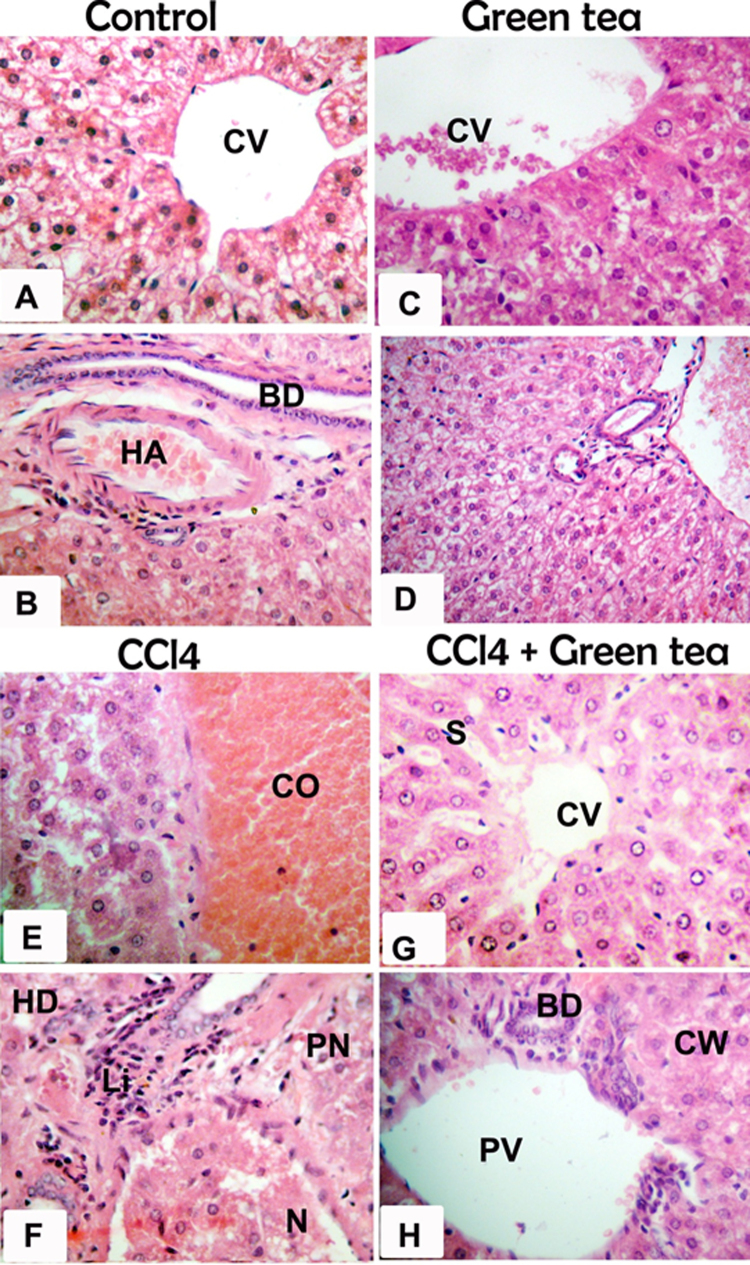
Photomicrographs of sections in liver of hamster (H&E X400) showing the normal architecture in the central vein (CV) and the portal area including hepatic artery (HA) and bile duct (BD) of control group (A, B) and green tea extract (C, D). CCl4- treated hamster show loss of architecture, congested central vein (CO), focal area of hepatic necrosis (N), pyknotic nuclei (PN), hydropic degeneration (HD) and infiltration with mononuclear inflammatory cells (Li) (E, F). CCl4 & green tea-treated Hamsters showing nearly normal hepatic structure represented by central vein (CV), hepatic sinusoid (S), portal vein (PV) and bile duct (BD), with mild degenerative changes of hepatocytes represented by cloudy swelling (CW), minimal vacuolation and less disarrangement of hepatocytes (G, H).

**Fig. 2:**
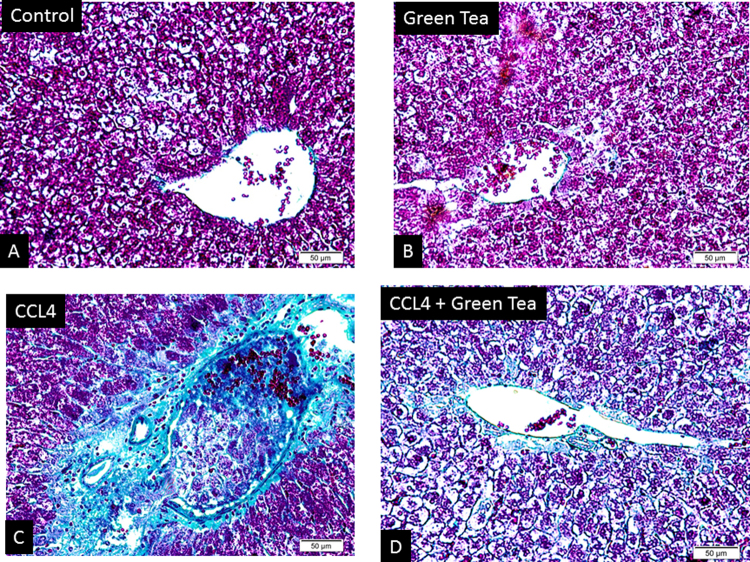
Histopathological changes of hepatic fibrosis occurred in CCl4-treated hamsters and prevention by the administration of green tea extract. (A) Normal control (B) CCl4 treatment (C) green tea extract (D) green tea extract + CCl4 (Masson trichrome stain).

The protective effect exerted by GTE against CCl4 induced-hepatotoxicity was confirmed by histological assessment (Fig. 1). CCl4 treated group revealed extensive liver injuries characterized by moderate to severe hepatocellular hydropic degeneration, necrosis, fibrosis and mononuclear inflammatory leukocyte infiltrations (E and F). However, CCl4 treated hamsters with GTE markedly ameliorated the hepatic lesions (G and H), respectively. Histopathologic changes in fibrosis occurred in CCl4-treated hamster’s livers, and their prevention by treatment with green tea extract was observed, as shown in Fig. 2. The fibrotic tissues had a blue colour by Masson’s trichrome stain. In control animals, the liver sections showed normal hepatic cells without fibrosis (A). A trace of hepatocyte fibrosis was observed in the livers of green tea extract-treated hamster (B). The livers of hamsters that were treated with CCl4 showed extensive accumulation of thick fibrotic tissue, resulting in the formation of continuous fibrotic septa compared to the normal control (C). Severe hepatic fibrosis induced by CCl4 was substantially reduced by the administration of green tea extract (D).

### 3.6 Immunohistochemistry of p53

**Fig. 3:**
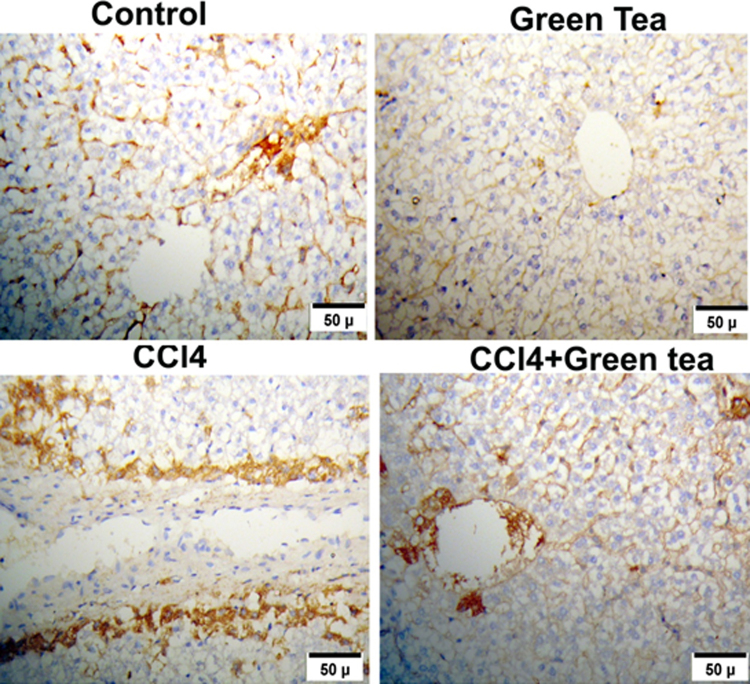
Impact of GTE on hepatic apoptosis induced- CCl4 by immunohistochemistry. A positive expression of p53 was found in CCl4 treated (C) compared to control (A) and GTE (B) treated hamsters. An improvement was shown in GTE co-administrated with CCl4 (D), a lower level of p53 expression was observed.

**Fig. 4:**
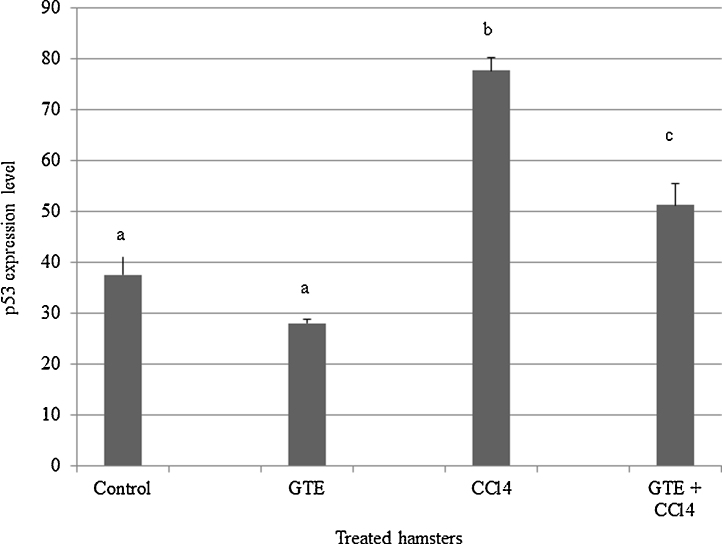
Level of p53 expression in liver of control, GTE, CCl4 and GTE+CCl4 treated hamsters. Positive proportions of p53 expression were increased significantly (*P* < 0.001) in liver of CCl4 treated hamsters compared to that in control and other treated groups.

A significantly (*P* < 0.001) higher expression of p53 was found in CCl4 treated group (C) compared to control (A) and GTE (B). An improvement was shown when GTE administrated with CCl4 (D), (Fig. 3). The supplementation of GTE in combination with CCl4 induced a significant (*P* < 0.05) reduction in p53 expression level (Fig. 4).

## 4 Discussion

Green tea extract was tested in this study for its ameliorating effect to CCl4 induced hepatic injury in male hamsters through assessment of body weight, liver weight, lipid profile, MDA, GSH, LDH and cytochrome P450 reductase as well as p53 expression. This study suggested that CCl4 treatment had no effect on body weight gain and relative liver weight. These results coincide with those of Uemitsu and Nakayoshi [51] who suggested no effect of oral CCl4 on liver weight. The increased level of MDA in CCl4 exposed hamsters is indicative for high level of lipid peroxidation which is in turn an indicator of membrane damage and alterations in structure and function of cellular membranes and failure of antioxidant defence mechanisms to prevent the formation of excessive free radicals [53]. Whereas CCl4 + GTE treated hamsters showed lower levels of MDA than CCl4-exposed hamsters, suggesting that GTE reversed the elevation of lipid peroxidation. Hence, it is possible that the mechanism of hepatoprotection of GTE may be attributed to the antioxidants present in GTE that scavenge a wide range of free radicals including most active hydroxyl radical which may initiate lipid peroxidation [18].

Results of current study demonstrated the potent effect of GTE in alleviation of CCL4 induced hepatic oxidative stress. This suggested that free radicals that released in the liver were effectively scavenged by green tea induced GSH that is because liver glutathione provide the first line of defence by scavenging ROS [22]. It appears that GSH conjugation is essential to decrease the toxic effects of CCl4. Elevation of GSH content by GTE observed in the present study may be due to the suppression of lipid peroxidation and protein oxidation [43].

Current results demonstrated highly significant (*P* < 0.001) reduction of hepatic ADH enzyme level in CCl4 treated group than control ones, while its level was significantly (*P* < 0.05) improved in GTE + CCl4 treated hamsters compared to CCl4 group. These results are in agreement with those of Park et al. [39] who confirmed the stimulatory effect of fermented green tea extract on mice hepatocytes induced toxicity model. ADH enzyme system serves in breakdown of alcohols, that are toxic, with the reduction of nicotinamide adenine dinucleotide into useful aldehyde, ketone, or alcohol groups during biosynthesis of various metabolites [15].

The data of the present work revealed a significant (*P* < 0.001) reduction in the level of cyotochrome P450 reductase in CCl4 treated hamsters. This could be attributed to the generating reactive oxygen species from CCl4 action on hepatocytes as well as lipid peroxidation that directly inactivate the enzyme and also labialize this enzyme for degradation by proteases [12], [20]. While the level of cyotochrome P450 reductase was improved in CCl4 + GTE treated hamster. This effect was abolished by GTE that ameliorated both oxidative stress and lipid peroxidation suggesting the positive effect of GTE in attaining normal hepatic function after induction of hepatotoxicity by CCl4.

Lipid profile alterations are a causal factor for excessive lipid peroxidation [32] and oxidative stress [50] that resulted from increase in ROS production and reduction in antioxidant enzymes, as demonstrated here by increased MDA and reduced GSH activities in CCl4 group. The effect of CCl4 could be reversed by GTE that ameliorated hepatotoxicity, lipid peroxidation and oxidative stress on hepatic tissue [34], [18].

In the present work, the influence of CCl4 and GTE on hepatic histology and p53 expression were studied. CCl4 treated hamsters showed extensive liver injuries characterized by moderate to severe hepatocellular hydropic degeneration, necrosis, fibrosis and mononuclear inflammatory leukocyte infiltrations. Hepatocyte fibrosis was evaluated by Masson’s trichrome stain. The detection of liver fibrosis often depends on the microscopic detection of collagen fibers, and Masson’s trichrome stain is a routine staining technique for detecting collagen fibers in liver tissue [5]. Hamsters that were treated with CCl4 + GTE showed less thick fibrotic tissue, which resulted in less pronounced destruction of the liver architecture compared to the CCl4 treatment group. Similar results in other studies confirmed that drinking water with green tea significantly decreased CCl4-induced hepatocyte fibrosis in rat livers [55]. According to the microscopic examinations, severe hepatic fibrosis induced by CCl4 was substantially reduced by the administration of green tea extract that were in accordance with the results of the oxidative damage analysis.

p53 expression was significantly (*P* < 0.01) increase in CCl4 treated hamsters than that in control ones. Interestingly, CCl4 + GTE treated hamsters markedly ameliorated the hepatic lesions and significantly (*P* < 0.05) decreased p53 expression than CCl4 treated hamsters. The histopathological observations in livers subjected to CCl4 are in agreement with the findings of previous studies [35], [30], while the ameliorating effect of green tea coincides with Hung et al. [23]. Severe hepatic fibrosis induced by CCl4 was substantially reduced by the administration of green tea extract, in accordance with the results of the oxidative damage analysis [49]. Zhen et al. [57] reported that green tea polyphenols arrest the progression of hepatic fibrosis in a rat model by inhibiting oxidative damage, as evidenced by decreased hydroxyproline levels in the rat livers suggesting that green tea extract has the ability to protect against CCl4-induced hepatic fibrosis. This could be due to GTE lowering effect of lipid peroxidation as lipid peroxidation increases the fluidity of membrane and consequently the inflammatory response in hepatic tissue [2]. Moreover, GTE could reduce lipid infiltration to hepatocytes, as observed here, because free fatty acids can play a crucial role in steatosis and necrotic-inflammatory processes intensification. Damage of lysosome integrity, which results in catepsin B and TNF-α release, is due to the effect of lipotoxicity can also occur. All these sequences lead to over expression of pro-apoptotic protein p53 that produced in response to hepatocytes inflammation [16], [38], [21]. P53 inhibits X-IAP induced cell death by promoting mitochondrial release Smac [8] thus resulting in apoptosis [14], [44], [42]. Thus GTE succeeded to manage the apoptotic and inflammatory effect of CCl4 on hepatocytes where the degree of hepatic injury was proven to be associated with a substantial number of hepatocytes undergoing apoptosis [24], [21]. This is might be the first to report the effect of CCl4 and GTE on p53 expression in hamsters.

## 5 Conclusion

CCl4-induced liver toxicity might be related to oxidative damage and lipid peroxidation. Drinking GTE has a beneficial effect decreasing lipid peroxidation, improving GSH, ADH, cytochrome P450 reductase activities and down-regulating p53 expression in hepatocytes. Further studies are needed to clarify the precise mechanisms of GTE action.

## Conflict of interests

The authors declare that they have no conflict of interests.
